# COVID‐19 reinfection in a healthcare worker after exposure with high dose of virus: A case report

**DOI:** 10.1002/ccr3.4257

**Published:** 2021-06-23

**Authors:** Shahram Ahmadian, Hadis Fathizadeh, Saeid Shabestari Khiabani, Mohammad Asgharzadeh, Hossein Samadi Kafil

**Affiliations:** ^1^ Faculty of Medicine Imam Reza Hospital Tabriz University of Medical Sciences Tabriz Iran; ^2^ Department of laboratory sciences Sirjan School of Medical Sciences Sirjan Iran; ^3^ Research Center for Pharmaceutical Nanotechnology Tabriz University of Medical Sciences Tabriz Iran; ^4^ Biotechnology Research Center Tabriz University of Medical Sciences Tabriz Iran; ^5^ Drug Applied Research Center Faculty of Medicine Tabriz University of Medical Sciences Tabriz Iran

**Keywords:** case report, COVID‐19, pandemic, reinfection, SARS‐CoV‐2

## Abstract

Reinfection with COVID‐19 is possible after exposure to a high dose of the virus. Due to immunity acquired during the previous infection, light symptoms are expected. The finding indicates importance of continuous protection in healthcare workers.

## Graphical abstract

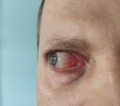

## 1 INTRODUCTION

Decreasing immunity happens in patients who have recovered from SARC‐CoV‐2. However, it remains unclear whether reinfection occurs. We encountered a case of reinfection of COVID‐19 caused re‐exposure to SARS‐CoV‐2 in a 36‐year‐old healthcare worker without obvious immunodeficiency. This case highlights the importance of the possibility of reinfection with SARS‐CoV‐2.

The outbreak of COVID‐19 disease caused by SARS‐CoV‐2, which started in Wuhan, China, in December 2019, has spread rapidly around the world and has now become a major health concern.^1^, ^2^ Fever, headache, shortness of breath, cough, fatigue, sore throat, vomiting and diarrhea, and loss of taste or smell have been reported as the most common symptoms of COVID‐19 disease.^3^, ^4^ Most cases are mild, but this disease is more severe in 14% of patients, and about 5% of patients need to be admitted to the intensive care unit (ICU).^5^, ^6^ Respiratory droplets caused by talking, sneezing, or coughing as well as contact are the most common routes of transmission. Patients with COVID‐19 and asymptomatic carriers and those in the incubation period are considered the main sources of infection.^1^, ^7^ The exact pathophysiological mechanism of SARS‐CoV‐2 has not yet been determined. In the current crisis, researchers have focused on clinical features, epidemiological history, effective drug discovery, and rapid detection methods. Therefore, less attention is paid to follow‐up and subsequent visits of the improved patients. Based on the available information about other coronaviruses, the disease caused by SARS‐CoV‐2 was also expected to induce a monophasic disease with at least a transient immunity, but rare cases of recurrence and reactivation of COVID‐19 have been reported.^8^, ^9^, ^10^ Here, we describe a case of reinfection of COVID‐19 after convalescence and re‐exposure with SARS‐CoV‐2.

## 2 CASE PRESENTATION

**FIGURE 1:**
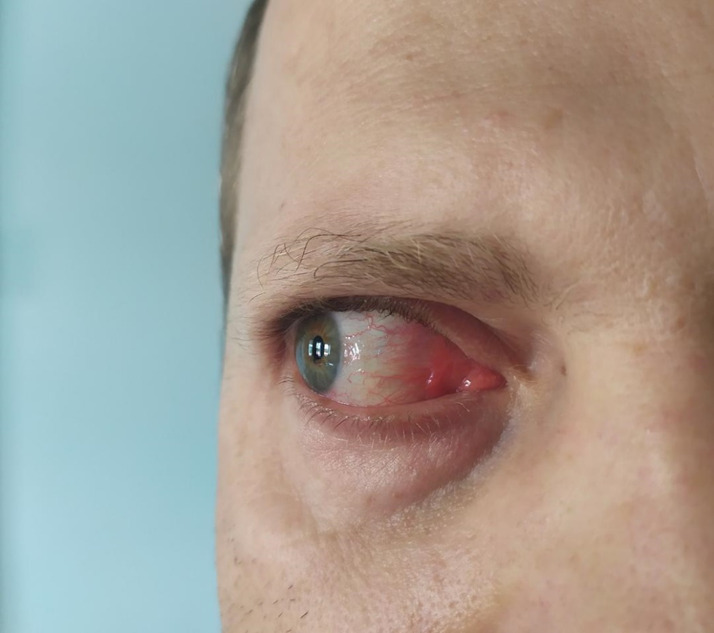
Redness and inflammation of the eye after exposure to the SARS‐CoV‐2

On June 21, 2020, a 36‐year‐old man in charge of collecting samples at the Corona Diagnosis Center in Tabriz, Iran, was evaluated for symptoms of lethargy and fatigue. On day 3 after symptom onset, he was tested positive for SARS‐CoV‐2 by qualitative real‐time reverse transcriptase‐polymerase chain reaction assay (qRT‐PCR) of the ORF1N gene (BioGerm) in nasopharyngeal swab according to WHO interim guidance.^11^ The patient did not report any underlying medical conditions such as diabetes, hypertension, or cardiovascular disease. CT scan showed mild pulmonary congestion. Also, he had CRP positive and blood changes such as lymphocyte depletion were observed. Two days later, he presented more severe shortness of breath, headache, fever, and chills. The patient was prescribed azithromycin and naproxen. Five days after the onset of the disease, a mild sore throat with occasional dry coughs was added to the previous symptoms. The disease lasted until July 4, when all symptoms disappeared completely and after which the patient recovered. On the fourteenth day, RT‐PCR was retested with a negative result. On August 25, when sampling a patient, coughing droplets sprayed on the face and eyes of this healthcare worker and caused redness, inflammation, and eye infection (Figure 1). The patient uses chloramphenicol drops and artificial tears, but after 4 days there was no change in redness and inflammation of the eye. On the fourth day, sampling of the eye and nasopharyngeal was performed and this time the result of RT‐PCR was positive for both ORF1ab and N. High viral load was also reported in the patient's eye. From August 29, symptoms began, including fatigue, fever, shortness of breath, and muscle pain in the femur and lower back. But all of these symptoms were much milder than the first episode of the disease. This time, there was no change in the patient's lymphocyte and neutrophil count, and the lymphocyte count was 36%, but the CRP was double positive. The patient was treated with azithromycin, acetaminophen, baclofen, gabapentin, and fluorometholone eye drops, and after about 10 days, he recovered.

## 3 DISCUSSION AND CONCLUSION

The rapid spread of SARS‐CoV‐2 has created significant health and economic challenges for many countries.^12^ The spectrum of COVID‐19 ranges from mild to life‐threatening. Some patients may progress quickly to acute respiratory distress syndrome (ARDS) and the involvement of several organs.^13^ On other hand, no definitive cure for this disease is known yet.^14^ Healthcare workers have the greatest risk of COVID‐19 contracting. During the SARS outbreak in 2002, 21 percent of those infected were healthcare workers.^15^ An important question that still remains unanswered is whether individuals who recover from this disease can be reinfected with the SARS‐CoV‐2? We reported a healthcare worker who had been infected once and recovered but showed symptoms again after re‐exposure to high volumes of the SARS‐CoV‐2. The number of such case reports is rare. In one study, of the 262 patients discharged from the hospital, 38 (14.5%) of them were tested positive for PCR again. However, these patients did not have clear clinical symptoms and disease progression.^16^ To et al examined reinfection of SARS‐CoV‐2 in an immunocompromised patient. The second phase was asymptomatic infection that occurred about 142 days after the initial symptomatic infection. They analyzed the genomic sequences of SARS‐CoV‐2 isolated from two episodes of COVID‐19 infection. Clinical, serological, epidemiological, and genomic analyses confirmed that this patient had reinfection instead of persistent viral shedding from the first infection. They suggest SARS‐CoV‐2 may maintain to circulate between the community populations despite herd immunity due to vaccination or natural infection.^17^ The results of a case report also indicate that reinfection could occur, although this study did not perform analysis of viral genome sequencing.^10^ Such case reports have increased discussion between reinfection and persistent SARS‐CoV‐2 shedding.^18^ In turn, Deng et al, in a study of 4 rhesus macaques based on radiological, pathological, and virology follow‐up stated that primary infection with SARS‐CoV‐2 could protect against reinfection, which is very important for vaccine design.^19^ However, neutralizing antibody increases quickly after primary infection, recent studies showed that titers of antibody start to decrease as early as 1‐2 months after the acute infection.^20^, ^21^, ^22^ Due to viral shedding to the detection limit point of RT‐PCR, sometimes patients whose test results are negative and discharged from the hospital having a recurrence of positive results.^23^ The available information states that shedding of SARS‐CoV‐2 may continue 20‐22 days after symptom onset and in some cases exhibiting shedding as long as 44 days.^24^, ^25^, ^26^ In one case series, 5‐13 days after the negative test result, patients were resampled and the results of the RT‐PCR test were positive again.^24^ Our patient had the first positive RT‐PCR test on the third day after the onset of symptoms and then had a negative swab on days 14 of the onset of illness in conjunction with symptomatic recovery. About 2 months later, due to nonobservance of safety tips and not wearing protective glasses, he became infected with the cough drops of another patient who was being sampled. The results of the person who infected this healthcare worker showed a very high load of the virus in the throat. Therefore, our patient became reinfected with a very large volume of SARS‐CoV‐2 that was spread in his eyes. There are some important points in our case report: (a) Second episode of COVID‐19 infection occurred due to receiving a very high number of viruses. (b) Despite the high load of SARS‐CoV‐2 in the eye and throat samples, the severity of disease was lower than the first infection and all symptoms were mild. (c) In the second episode, the period of COVID‐19 infection was shorter and recovery was faster. (d) Most importantly, RT‐PCR assay result was positive for both N and RdRp genes in the second episode of COVID‐19, while was just positive for the N gene in the first episode of disease. Probably, the strains of SARS‐CoV‐2 that this healthcare worker was infected with them were genetically different due to high ability of genetic mutations creating of SARS‐CoV‐2. Taken together, cases of reinfection or reactivation of SARS‐CoV‐2 have been reported, despite differences in the data provided, including serological data, reactivation time, and disease period, patients who tested positive again had generally milder disease or asymptomatic course.^27^ There were some of limitations in this study: first, lack of serological analysis of patient serum in two episodes of disease to assay antibody levels and second, the impossibility of sequencing of SARS‐CoV‐2 isolated of this case. Here, we discussed a case of possible COVID‐19 reinfection. This case demonstrated that reinfection can occur even just after a few months of recovery from the first infection. Future studies of patients with reinfection could provide a better understanding of the pathogenic behavior and genetic nature of the SARS‐CoV‐2 and help to the design an efficiency vaccine to provide immunity to the COVID‐19 infection. Another more important issue is the need to preventive measures such as the use of masks and social distancing, even for those who have been infected once.

## CONFLICT OF INTEREST

None to declare.

## AUTHOR CONTRIBUTION

SA: involved in sample collection and manuscript preparation. HF: involved in sample collection, RNA extraction, and manuscript preparation. SK: involved in data annotation. MA: involved in data analysis and manuscript preparation. HK: involved in RNA extraction, RT‐PCR analysis, data analysis, data annotation, and manuscript preparation. All authors have read and approved the manuscript.

## ETHICAL APPROVAL

This study was approved in local ethic committee with reference number IR.TBZMED.REC.1399.665 during local meeting at 2020.09.29. Link is available at https://ethics.research.ac.ir/IR.TBZMED.REC.1399.665. This study had no funding and consent forms are gotten for eligibility of publishing figure of the eye and data sharing of the patients and are available upon request. All ethical issues were followed according to Helsinki declaration.

## CONSENT FOR PUBLICATION

Written informed consent was obtained from the patient for publication of this case report and any accompanying images. A copy of the written consent is available for review by the Editor‐in‐Chief of this journal.

## Data Availability

All analyses related to the study are available upon sending request to the corresponding author.
